# The extremity localized classic osteosarcomas have better survival than the axial non-classics

**DOI:** 10.1186/s12957-018-1344-3

**Published:** 2018-02-23

**Authors:** Li Lin, Shaoyong Deng, Futing Zhang, Yaoze Liang, Zhenhua Huang

**Affiliations:** 1 Department of Oncology, Nanfang Hospital, Southern Medical University, Guangzhou, 510515 China; 2 Department of 7th Surgery, Guangdong 999 Brain Hospital, Guangzhou, 510510 China; 3 Department of Medical Treatment Quality Management, Nanfang Hospital, Southern Medical University, Guangzhou, 510515 China

**Keywords:** Osteosarcoma, Overall survival, Event-free survival, Extremity, Axial

## Abstract

**Background:**

Osteosarcoma is one of the most malignant primary bone cancers, while is rarely reported in China. Of note, very few data of prognosis has been documented in this region. Thus, we carried a retrospective study to identify prognostic factors and to analyze outcomes in patients of both classic and non-classic high-grade osteosarcomas. Classic osteosarcoma is defined as of high-grade histology, age below 40 years, with extremity localized primary tumor, and without detectable metastasis at primary diagnosis.

**Methods:**

A total of 98 patients (68 classic and 30 non-classic) aged from 4 to 64 years old were diagnosed as high-grade osteosarcoma from 2008 to 2015 in Nanfang Hospital, Guangzhou, China. Univariate and multivariate analyses were performed to identify the independent predictors for overall survival and event-free survival. Kaplan-Meier method was used for survival analysis.

**Results:**

The median overall survival was 117 vs. 21 months, and the median event-free survival was 31 vs. 6 months in classic and non-classic osteosarcoma, respectively. The most frequently found tumor site was around the knee. The classic osteosarcoma had better overall survival and event-free survival than the non-classics. Tumor site and primary metastasis were found to be associated with overall survival and event-free survival in the univariate analysis. In the multivariate Cox regression analysis, tumor site and primary metastasis were each verified as independent prognostic factors. However, no similar result was found in elevated serum alkaline phosphatase or lactate dehydrogenase. Amputation or limb salvage surgery had no significant effect on overall survival and event-free survival in the extremity osteosarcomas. Classic osteosarcomas with extremity tumor site and free of primary metastasis exhibited better overall survival and event-free survival, while the axial and metastatic non-classics exhibited the worse.

**Conclusions:**

The extremity classic osteosarcomas have better survivals than the axial non-classic cases. Amputation and limb salvage surgery make no significant change in overall survival and event-free survival in the extremity osteosarcomas.

**Trial registration:**

Nanfang2013071; Date of registration: 7 September 2013 (retrospectively registered).

## Background

Osteosarcoma is one of the most malignant human primary bone cancers, which mainly affects children and adolescents with a median age of about 20 years old. The incidence rate is rare in China with only about 3 per million persons every year. This fact is probably the reason why few data of osteosarcoma was documented or analyzed referring to the prognosis in this region.

Advances in the clinical management of osteosarcoma, specifically the introduction of multiagent chemotherapy in combination with surgery, had led to dramatic prognostic improvements in 5-year survival rates from less than 20% to 55–80% in the 1980s [1, 2]. Several international studies have demonstrated that MAP (high-dose methotrexate, doxorubicin, and cisplatin) regimen was the most effective chemotherapy against osteosarcoma [3–5]. However, additional improvements in survival rates have not been achieved in the last 30 years [6], and there are still controversies in some aspects concerning prognostic evaluation and therapeutic approaches [2, 7–21]. For example, it remains unknown whether elevated serum alkaline phosphatase (ALP) or lactate dehydrogenase (LDH) can be regarded as independent prognostic factors. In the extremity osteosarcomas, no consensus has been reached on whether patients can benefit from amputation or limb salvage surgery. Furthermore, very limited information is known about survival and prognostic factors in patients across all subgroups of osteosarcomas because most of the previous studies predominantly focused on “classic osteosarcoma”, which should merit the whole criteria of age < 40 years old, high-grade histology, extremity localized primary tumor, and no detectable metastasis at primary diagnosis [22]. The “non-classic osteosarcoma” was defined as primary metastatic disease, non-extremity localization, or age > 40 years [22].

Therefore, we conducted a retrospective study covering both classic and non-classic osteosarcoma in our center, which is located in the South China. The aim of this study is to find out the prognostic factors of osteosarcoma and to analyze the overall survival (OS) and event-free survival (EFS) in different patient subgroups.

## Methods

### Patients

From January 2008 to December 2015, patients histologically diagnosed as high-grade osteosarcoma were treated at Nanfang Hospital, Southern Medical University, Guangzhou, China. Inclusion criteria were as follows: (1) patients treated with neoadjuvant chemotherapy, surgery, and adjuvant chemotherapy and (2) patients with chemotherapy protocols containing MAP (high-dose methotrexate, doxorubicin, and cisplatin) regimen. Patients who received less than 6 months of chemotherapy and discontinued therapy without disease progression or death were excluded from this study, while those who reached death or progression of disease were included in this study. In total, 98 patients that merit the criteria were enrolled and followed up. Survival status was ascertained through examination in the outpatient clinic or telephone interviews. This study was approved by the Nanfang Hospital Ethics Review Board with the consent of each patient.

### Diagnosis and treatments

All osteosarcoma diagnoses were confirmed by histopathology. All patients followed neoadjuvant chemotherapy, definitive surgery, and adjuvant chemotherapy. The types of surgery for extremity tumors were either limb salvage surgery or amputation. Surgeries of the axial osteosarcomas were limited to tumor conservative resection. All detectable metastatic sites were also removed surgically, whenever possible. Both neoadjuvant and adjuvant chemotherapies adopt the uniform MAP regimen, including high-dose methotrexate (8 to12 g/m^2^ per course with leucovorin rescue), doxorubicin (90 mg/m^2^ per course), and cisplatin (75 to 90 mg/m^2^ per course). When diseases progressed, ifosfamide, etoposide, cyclophosphamide, vincristine, paclitaxel, and dacarbazine were used in various combinations as next-line chemotherapies. The total duration of chemotherapy ranged from 6 to 10 months.

### Assessed variables

Gender, age, duration of symptoms, primary tumor site, tumor size, primary metastasis status at diagnosis, serum alkaline phosphatase (ALP), lactate dehydrogenase (LDH), type of surgery, and outcome data were collected retrospectively from clinical records. An age of 40 years was used as the cut-off point in prognosis analysis as in the study of COSS [23]. Axial sites include the head and neck, trunk, clavicles, and pelvis bone. Tumor size was defined as 8 cm or less (small) and greater than 8 cm (large) [24]. Duration of symptoms means the duration from symptoms’ occurrence to pathological diagnoses, and the median time was used as cut-off point. Reference values of serum ALP and LDH are 45–125 U/L and 0–248 U/L, respectively.

### Statistical analysis

Data was censored on March 31, 2016. Descriptive statistics were presented with mean ± SD or median for continuous variables and percentages for categorical variables. Univariate survival analysis was carried out using Kaplan-Meier method and compared by the log-rank test. Multivariate Cox-regression analysis was used to identify independent prognostic factors [2, 23, 25, 26]. Chi-square test was used for comparison of categorical variables and independent samples’ *T* test (normal distribution) or Mann-Whitney *U* test (abnormal distribution) for continuous variables. ANOVA (analysis of variance) was used to test the difference of the mean of two or more samples. OS was calculated from the date of diagnosis to the date of death from any cause or last visit. EFS was calculated from the date of diagnosis to the date of disease relapse, or progression, or death from any cause, whichever occurred firstly, or last visit. All statistical analyses were carried out using SPSS statistics (version 22). Statistical significance was defined as *P* ≤ 0.10 in univariate analysis and *P* < 0.05 in other analysis.

## Results

### Patient characteristics

**Fig. 1:**
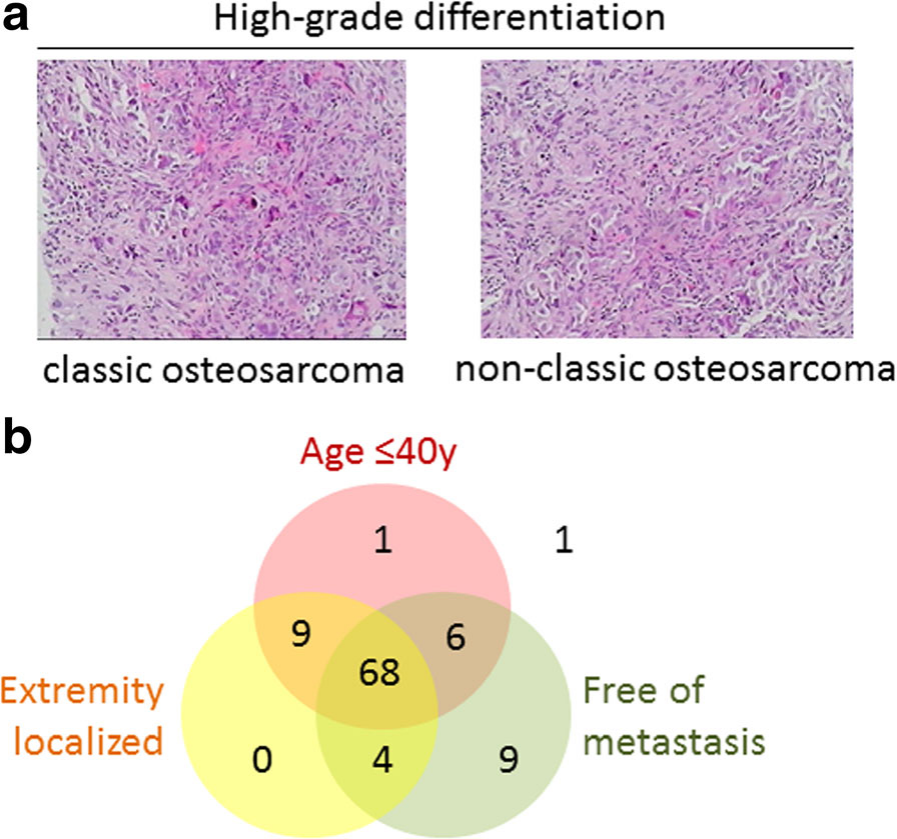
**a** Representative hematoxylin-eosin staining of classic and non-classic high-grade osteosarcomas in this study. **b** Schematic figure showing the distribution of classic and non-classic high-grade osteosarcoma patients of this study. The three intersecting circles comprised age ≤ 40 years, extremity localized primary tumor, or free of metastasis at primary diagnosis, to illustrate the overlapping parts between the three elements of the definition of classic osteosarcoma

From 2008 to 2015, 98 patients were histologically diagnosed as osteosarcoma and received treatments in our center. Since all included patients were of high-grade differentiation, no great histological difference was found between the classic osteosarcoma and the non-classics in this study (Fig. 1a). Among them, 84 patients were under 40 years, 81 patients were extremely localized, and 87 patients were free of metastasis. The intersection of these three groups was defined as classic osteosarcoma, including 68 (69.38%) patients. The rest 30 patients were diagnosed as non-classic osteosarcoma, among which 19 merited two of the aforementioned criteria, 10 merited only one criterion, and 1 did not belong to any of the three standards (Fig. 1b). There were 60 male patients (61.2%; median age, 18 years; range, 7 to 62 years) and 38 female patients (38.8%; median age, 15 years; range, 4 to 64 years). The median onset age was 18 years (range, 4 to 64 years; mean, 23.06 ± 1.530 years). Patients aged ≤ 40 years accounted for about 85.71% of all the patients. Half of the total patients were attacked in the second decade of life, while it was 58.8% in the classic group. When the first symptoms occurred, 62.2% of the patients visited doctors and were diagnosed within 2 months. The most frequent primary sites were around the knee (55.1%), including the distal femur (35.7%), proximal tibia (12.2%), and proximal fibula (7.1%). The proximal humerus, the second most common site of osteosarcoma reported in another study [23], accounted for 6.1% of cases in our study, while the axials are of 17.3%. Referring to tumor size, tumors of 60 patients (61.2%) were with diameter ≤ 8 cm. Therein, it seems that non-classic sarcoma patients tend to have a smaller tumor size than the classics, yet no statistical significance was reached by Chi-square test (70.0 vs. 57.4%, *P* = 0.236). Eleven of 98 patients presented with distant metastases at first diagnosis, and the lung is the major site of metastasis (Table 1).

**Table 1:** Baseline and treatment characteristics

Characteristic	Classic	Non-classic	Total
Gender			
Male	43	17	60 (61.2%)
Female	25	13	38 (38.8%)
Age			
0–10	13	1	14 (14.3%)
11–20	40	9	49 (50.0%)
21–30	10	1	11 (11.2%)
31–40	5	5	10 (10.2%)
41–50	–	4	4 (4.1%)
51–60	–	6	6 (6.1%)
> 60	–	4	4 (4.1%)
Duration of symptoms			
≤ 2 months	45	16	61 (62.2%)
> 2 months	23	14	37 (37.8%)
Tumor site			
Around knee	46	8	54 (55.1%)
Distal femur	31	4	35 (35.7%)
Proximal tibia	9	3	12 (12.2%)
Proximal fibula	6	1	7 (7.1%)
Proximal humerus	5	1	6 (6.1%)
Other extremities	17	4	21 (21.4%)
Axial	–	17	17 (17.3%)
Tumor size			
≤ 8 cm	39	21	60 (61.2%)
> 8 cm	29	9	38 (38.8%)
Primary metastasis			
No	68	19	87 (88.8%)
Yes	–	11	11 (11.2%)
Lung	–	9	9 (9.2%)
Distal bones	–	1	1 (1.0%)
Other sites	–	1	1 (1.0%)
Serum ALP			76 available
Normal	20	7	27 (35.5%)
>ULN	38	11	49 (64.5%)
Serum LDH			52 available
Normal	25	12	37 (71.2%)
>ULN	14	1	15 (28.8%)
Type of extremity surgery			81 available
Limb salvage	28	10	38 (46.9%)
Amputation	40	3	43 (53.1%)

*ALP* alkaline phosphatase, *LDH* lactate dehydrogenase, *ULN* upper limit of normal

It was suggested that serum ALP and LDH were two laboratory examinations that may elevate in osteosarcoma. Therefore, we analyzed the serum ALP and LDH levels at primary diagnosis, which were totally documented in 76 and 52 patients, respectively. Among them, elevated ALPs were examined in more than half of the patients, while elevated LDHs were found in less than one third. All the 81 extremity osteosarcoma patients received either limb salvage surgery (38 patients) or amputation (43 patients) (Table 1).

### The classic osteosarcomas have better OS and EFS than the non-classics

**Fig. 2:**
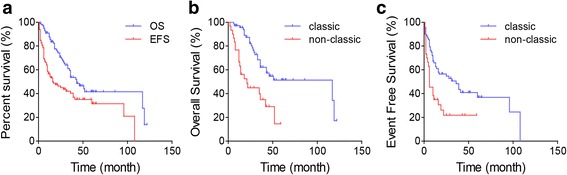
**a** Survival curves of OS and EFS in all osteosarcoma patients in this study. **b** OS and **c** EFS curves of the classic and non-classic osteosarcomas.

Median follow-up was 29 months (range, 2 to 122 months) for all 98 patients and 39 months (range, 8 to 122 months) for 51 survivors. The median OS was 43 months and median EFS was 16 months. OS rates at 2 and 5 years were 70.5 ± 4.8% and 41.5 ± 6.3%, respectively. EFS rate at 2 and 5 years were 46.2 ± 5.2% and 31.5 ± 5.9%, respectively (Fig. 2a). Among these patients, it is obvious that classic osteosarcoma had better prognosis than the non-classics (Mann-Whitney *U* test; *P* = 0.001 for OS, *P* = 0.001 for EFS) (Fig. 2b, c). The 2-year and 5-year OS rates of classics vs. non-classics were 82.1 ± 4.9% vs. 44.9 ± 9.3% and 51.4 ± 7.4% vs. 14.6 ± 11.4%, while the 2-year and 5-year EFS rates were 56.4 ± 6.1% vs. 21.8 ± 8.2% and 36.8 ± 7.2% vs. 21.8 ± 8.2%, respectively.

### Primary tumor location and metastasis status are independent prognostic factors for osteosarcoma

Based on the aforementioned clinical features, we hope to find out the prognostic factors for osteosarcoma OS and EFS. Of note, to the parameter of surgery type, we only enrolled the extremity osteosarcoma patients with limb salvage surgery or amputation, while the axials were excluded. As the results of the univariate survival analysis by Kaplan-Meier method, axial tumor site (OS, *P* = 0.000; EFS, *P* = 0.000) and the presence of primary metastasis (OS, *P* = 0.001; EFS, *P* = 0.042) were associated with inferior OS and EFS. Whereas, there were no significant correlations between outcome and factors including gender, age, tumor size, duration of symptoms, serum ALP, or serum LDH in this study. In the extremity osteosarcomas, there was no OS or EFS difference between amputation and limb salvage (Table 2).

**Table 2:** Univariate analysis for OS and EFS

Variable	OS		EFS	
	Median	*P*	Median	*P*
Gender				
Male	43	0.735	14	0.478
Female	43		16	
Age				
≤ 40 years	46	0.101	22	0.116
> 40 years	18		6	
Duration of symptoms				
≤ 2 months	46	0.888	16	0.891
> 2 months	36		17	
Tumor site				
Extremity	51	<0.001	25	<0.001
Axial	14		5	
Tumor size				
≤ 8 cm	52	0.798	17	0.920
> 8 cm	36		16	
Primary metastasis				
No	51	0.001	25	0.042
Yes	14		10	
Serum ALP				
Normal	51	0.871	13	0.595
>ULN	52		22	
Serum LDH				
Normal	36	0.671	9	0.165
>ULN	NA^a^		NA^a^	
Type of extremity surgery				
Limb salvage	46	0.869	22	0.606
Amputation	NA^a^		9.5	

*OS* overall survival, *EFS* event-free survival, *ALP* alkaline phosphatase, *LDH* lactate dehydrogenase, *ULN* upper limit of normal

^a^The median OS and EFS are not available in >ULN LDH group, and median OS is not available in amputation surgery group because > 50% patients were free of events

Then, we performed multivariate Cox-regression analysis to figure out the independent prognostic factors for osteosarcoma OS and EFS. Variables of primary tumor site and metastasis status, which had a statistical significance in the univariate analysis, were evaluated. In addition, the variable of age, which had a marginal *P* value in both OS and EFS, was also enrolled. Moreover, variables of tumor size and type of surgery, the prognostic values of which were controversial in previous studies, were also analyzed. The multivariate analysis revealed that tumor site and primary metastasis were independent prognostic factors for both OS and EFS in osteosarcoma patients. Extremity site and lack of primary metastasis presented improved trends in OS and EFS. No significance was reached in age, tumor size, and type of surgery (Table 3).

**Table 3:** Multivariate analysis for OS and EFS

Variable (Category)	OS			EFS		
	HR	95% CI	*P*	HR	95% CI	*P*
Age (≤ 40 year vs. > 40 year)	1.075	0.393–2.946	0.888	0.959	0.404–2.280	0.925
Tumor site (limb vs. axial)	0.221	0.080–0.609	0.004	0.286	0.123–0.666	0.004
Tumor size (≤ 8 cm vs. > 8 cm)	0.783	0.410–1.494	0.457	0.886	0.510–1.540	0.668
Metastasis (no vs. yes)	0.307	0.142–0.661	0.003	0.441	0.207–0.938	0.034
Surgery (salvage vs. amputation)	0.889	0.445–1.777	0.738	0.730	0.394–1.353	0.318

*HR* hazard ratio, *CI* confidence interval, *OS* overall survival, *EFS* event-free survival

### The extremity localized and metastasis-free classic osteosarcomas have longer OS and EFS

**Fig. 3:**
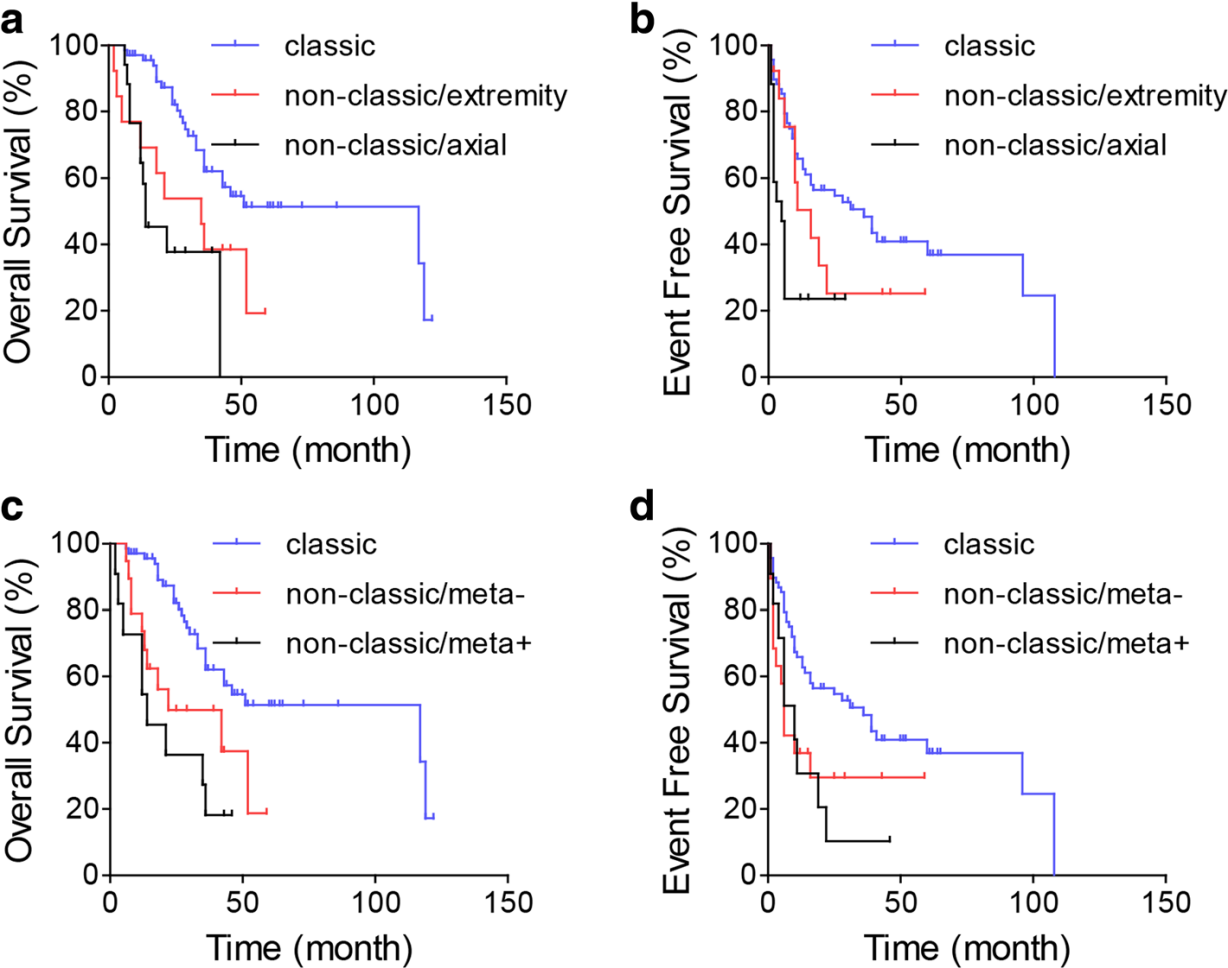
Survival curves of **a**, **c** OS and **b**, **d** EFS in the osteosarcomas. Patients are categorized by classic or non-classic combined with (**a** and **b**) primary tumor location or (**c** and **d**) metastatic status

Since the classic osteosarcoma had better OS and EFS than the non-classics, we want to know whether the extremity localized and metastasis-free classic osteosarcomas are superior to the extremity or axial non-classics and metastatic or metastasis-free non-classics. The survivals of extremity localized classic osteosarcomas and extremity or axial non-classic osteosarcomas were analyzed by ANOVA. The result showed that the extremity site classic osteosarcomas had superior survival comparing to the axial non-classic osteosarcomas in both OS (*P* = 0.003) and EFS (*P* = 0.004) (Fig. 3a, b)*.* Among the metastasis-free classic, metastasis-free non-classic, and metastatic non-classic subgroups, metastasis-free classic osteosarcomas had the best OS (*P* = 0.036) and EFS (*P* = 0.054) (Fig. 3c, d).

## Discussion

Osteosarcoma is a kind of solid tumor with relatively low incidence, which up to now lacks clinical documentary. Most of the previous studies only referred to the classic osteosarcomas, while few articles have focused on the prognosis of the non-classics. The present study enrolled both classic and non-classic osteosarcoma patients with histologically proven high-grade, and all the enrolled patients were treated with the uniform strategies with neoadjuvant chemotherapy (MAP regimen), surgery, and adjuvant chemotherapy (MAP regimen). The uniform treatment eliminates the bias associated with therapeutic regimen, making comparisons valid.

Our survival results are similar to those in other previous studies. A single-institution study from Malaysia recruiting 163 patients reported 5-year OS of 44% and 5-year DFS (disease-free survival) of 33% [27]. The Brazilian osteosarcoma treatment group studies III and IV showed that 5-year OS was 50.1% and 5-year EFS was 39% [13]. An American team using Surveillance, Epidemiology, and End Results (SEER) program database to analyze the survival in 2849 patients with high-grade osteosarcoma presented 5-year OS of 71.8% in non-metastatic disease and 30.4% in metastatic disease at diagnosis [28]. Collectively, the reviewed studies showed that 5-year OS and EFS ranged from 37.9 to 77% and 38 to 62%, respectively [2, 7–21]. Consistent with these previous studies, the survival data in this study were located within the same range as reported before.

In this study, we enrolled primary tumor site and metastasis status at diagnosis, as well as gender, age, tumor size, symptom duration, type of surgery, and serum ALP and LDH levels for prognostic analysis, some of which have also been analyzed in other previous studies, but some of the results are still controversial. In Table 4, we listed some similar studies as ours.

**Table 4:** Review of prognostic factors for OS and/or EFS in previous studies by univariate and multivariate analysis

	Vasquez et al. [9]		Whelan et al. [2]		Pakos et al. [15]		Janeway et al. [17]		Pruksakorn et al. [18]		Duchman et al. [28]		Min et al. [8]		Faisham et al. [27]		Petrilli et al. [13]	
	*n* = 73		*n* = 1067		*n* = 2680		*n* = 1054		*n* = 144		*n* = 2949		*n* = 333		*n* = 163		*n* = 209	
	OS + EFS		OS + PFS		OS		OS + EFS		OS		OS		OS		OS		OS + EFS	
	UVA	MVA	UVA	MVA	UVA	MVA	UVA	MVA	UVA	MVA	UVA	MVA	UVA	MVA	UVA	MVA	UVA	MVA
Gender	ns	–	s	s	ns	–	ns	–	ns	–	s	s	s	s	s	ns	–	–
Age	ns	–	ns	–	s	s	s	s	s	ns	s	s	ns	–	s	s	–	–
Tumor site	ns	–	s	s	s	s	s	s	s	s	s	s	ns	–	–	–	–	–
Tumor size	s	ns	–	–	–	–	–	–	ns	–	s	s	–	–	–	–	s	ns
Symptom duration	–	–	–	–	–	–	–	–	ns	–	–	–	–	–	–	–	–	–
Primary metastasis	s	s	–	–	s	s	s	s	s	s	s	s	–	–	s	s	s	s
Serum ALP	s	s	–	–	–	–	–	–	–	–	–	–	s	s	–	–	–	–
Serum LDH	ns	–	–	–	–	–	–	–	–	–	–	–	–	–	–	–	–	–
Surgery type	–	–	s	ns	s	s	–	–	–	–	–	–	ns	–	–	–	s	–

*s* significant, *ns* not significant, *OS* overall survival, *EFS* event-free survival, *PFS* progression-free survival, *UVA* univariate analysis, *MVA* multivariate analysis, *ALP* alkaline phosphatase, *LDH* lactate dehydrogenase

Our study showed that primary metastasis status at diagnosis was an independent prognostic factor. Seven of the 9 studies evaluated primary metastasis status, and all validated it as an independent prognostic factor for the outcome of osteosarcoma, which is in fact a foregone conclusion. The study of American SEER program database revealed primary metastasis as an independent predictor for cause-specific survival at 10 years [28]. Pakos et al. [15] showed that the death risk of metastasis at diagnosis is nearly three folds than that of non-metastasis, while Janeway et al. [17] reported that the presence of metastasis at diagnosis had a poorer survival in patients with high-grade osteosarcoma of any site enrolled on North American Cooperative Group. Similarly, the other four studies demonstrated that clinically detectable primary metastasis were significantly associated with inferior outcome [9, 13, 18, 27].

Primary tumor site was another independent osteosarcoma prognostic factor in our study, which was supported by most of the listed studies in Table 4 [2, 15, 17, 18, 28]. It is worth noting that Pruksakorn et al. [18], Duchman et al. [28], and Min et al. [8] used the same categorization as ours in the analysis of tumor site which compared extremity tumors with axial tumors. Consistent with our data, Duchman et al. [28] and Pruksakorn et al. [18] reported that axial osteosarcomas have lower survival rate than the extremities. With different categorizations, Whelan et al. [2] showed that patients of distal tumor location had better survival than those of proximal humerus/femur, Pakos et al. [15] showed that the death risk of tibia tumor location was lower than that of femur tumor location, and Janeway et al. [17] reported that pelvic tumor site was associated with a decrease in EFS and OS.

Other variables of gender, age, tumor size, duration of symptoms, type of surgery, and serum ALP and LDH levels analyzed in our study did not show predictive values for OS or EFS, and some coincident negative results can also be found in other studies (Table 4). These studies, except that of Petrilli et al. [13], evaluated gender in univariate analyses, but only four reported significant association with outcome, three of which suggested that female patients had a better outcome than male patients in the multivariate analyses. Hence, whether gender is an independent prognostic factor for patients with osteosarcoma remains controversial. There are eight of these studies evaluated the predictive value of age, and only four studies showed elder age was associated with worse outcome of osteosarcoma. However, it must be noted that they used different cut-off points, i.e., 18 years in the study of Janeway et al. [17] and 60 years in the study of Duchman et al. [28]. Converted to ranked data without cut-off points, elder age was reported as an adverse prognostic factor with 7% relative risk increasing per decade by Pakos et al. [15]. Faisham et al. [27] showed that patients older than 12 years had worse survival, but no significant difference was found when the cut-off point was 40 years. Referring to tumor size, only Duchman et al. [28] reported it as an independent risk factor which was divided into small (diameter ≤ 5 cm), intermedia (5–10 cm), large (> 10 cm), or unknown. Other three studies used different categorizations (i.e., diameter of 12 cm [9, 13] or tumor volume of 180 ml [18] as cut-off points) but obtained no significant result. Our study categorized tumor size by the diameter of 8 cm according to TNM Staging system [24], which was the same as the studies by Nathan and Healey [16] and Wang et al. [20], and we did not find significant difference between these two groups. Of these nine studies, only Prusakorn et al. [18] evaluated the symptom duration before diagnosis, and the result showed that it was not a predictor for the outcome of ostnon-classic osteosarcoma, which was the same as our data. There were few researches focusing on the effect of ALP or LDH. Only Vasquez et al. [9] examined the predictive value of LDH and found that it was not a prognostic factor. Vasquez et al. [9] and Min et al. [8] reported that raised ALP was associated with decreased prognosis. Type of surgery was evaluated by four studies, while only the data of Pakos et al. [15] revealed it as an independent prognostic factor, which showed that the risks of metastasis and death would increase in patients undergoing an amputation. Given the different conclusions reported in literature, we speculated that this might result from patient heterogeneity, different cut-off values, and small sample sizes in most of the studies. Therein, different demographic backgrounds, tumor-related characteristics, and therapy protocols account for the heterogeneity of patients. The subjects of these studies are of different races, gender proportions, and age distributions, which may also lead to varied characters of tumor genesis, progression, and outcome. These at least partly explain why most of the studies are limited to patients with classic osteosarcoma, which is the common form of osteosarcoma with almost uniform treatment.

## Conclusions

Classic osteosarcomas have better prognosis than non-classic osteosarcomas. The prognostic analysis shows that primary tumor site and metastasis status are independent predictive factors for OS and EFS, but no significance is reached in elevated serum alkaline phosphatase or lactate dehydrogenase. The extremity localized classic osteosarcomas have better survivals than the axial non-classic cases. Amputation or limb salvage surgery has no significant effect on OS and EFS in the extremity osteosarcomas.

## Abbreviations

ALP: Serum alkaline phosphatase
CI: Confidence interval
COSS: Cooperative osteosarcoma study
CT: Computed tomography
EFS: Event-free survival
HR: Hazard ratio
LDH: Lactate dehydrogenase
MAP: High-dose methotrexate, doxorubicin and cisplatin
MDP: Methylene diphosphonate
MRI: Magnetic resonance imaging
OS: Overall survival
PET/CT: Positron emission tomography/computed tomography
SD: Standard deviation
SEER: Surveillance, epidemiology, and end results
SPSS: Statistical product and service solutions
ULN: Upper limit of normal

## References

[1] Nataraj V, Batra A, Rastogi S, Khan SA, Sharma MC, Vishnubhatla S, Bakhshi S (2015). Developing a prognostic model for patients with localized osteosarcoma treated with uniform chemotherapy protocol without high dose methotrexate: a single-center experience of 237 patients. J Surg Oncol.

[2] Whelan JS, Jinks RC, McTiernan A, Sydes MR, Hook JM, Trani L, Uscinska B, Bramwell V, Lewis IJ, Nooij MA (2012). Survival from high-grade localised extremity osteosarcoma: combined results and prognostic factors from three European Osteosarcoma Intergroup randomised controlled trials. Ann Oncol.

[3] Link MP, Goorin AM, Horowitz M, Meyer WH, Belasco J, Baker A, Ayala A, Shuster J. Adjuvant chemotherapy of high-grade osteosarcoma of the extremity. Updated results of the Multi-Institutional Osteosarcoma Study. Clin Orthop Relat Res. 1991;270:8–14. 1884563

[4] Luetke A, Meyers PA, Lewis I, Juergens H (2014). Osteosarcoma treatment—where do we stand? A state of the art review. Cancer Treat Rev.

[5] Ferrari S, Serra M (2015). An update on chemotherapy for osteosarcoma. Expert Opin Pharmacother.

[6] Ritter J, Bielack SS (2010). Osteosarcoma. Ann Oncol.

[7] Durnali A, Alkis N, Cangur S, Yukruk FA, Inal A, Tokluoglu S, Seker MM, Bal O, Akman T, Inanc M (2013). Prognostic factors for teenage and adult patients with high-grade osteosarcoma: an analysis of 240 patients. Med Oncol.

[8] Min D, Lin F, Shen Z, Zheng S, Tan L, Yu W, Yao Y (2013). Analysis of prognostic factors in 333 Chinese patients with high-grade osteosarcoma treated by multidisciplinary combined therapy. Asia Pac J Clin Oncol.

[9] Vasquez L, Tarrillo F, Oscanoa M, Maza I, Geronimo J, Paredes G, Silva JM, Sialer L (2016). Analysis of prognostic factors in high-grade osteosarcoma of the extremities in children: a 15-year single-institution experience. Front Oncol.

[10] Berlanga P, Canete A, Diaz R, Salom M, Baixauli F, Gomez J, Llavador M, Castel V (2015). Presentation and long-term outcome of high-grade osteosarcoma: a single-institution experience. J Pediatr Hematol Oncol.

[11] Serlo J, Tarkkanen M, Sampo M, Vettenranta K, Riikonen P, Helenius I (2015). Incidence, treatment and survival of paediatric patients with bone sarcomas in Finland from 1991 to 2005. Acta Paediatr.

[12] Hung GY, Yen HJ, Yen CC, Wu PK, Chen CF, Chen PC, Wu HT, Chiou HJ, Chen WM (2016). Improvement in high-grade osteosarcoma survival: results from 202 patients treated at a single institution in Taiwan. Medicine (Baltimore).

[13] Petrilli AS, de Camargo B, Filho VO, Bruniera P, Brunetto AL, Jesus-Garcia R, Camargo OP, Pena W, Pericles P, Davi A (2006). Results of the Brazilian Osteosarcoma Treatment Group Studies III and IV: prognostic factors and impact on survival. J Clin Oncol.

[14] Hegyi M, Semsei AF, Jakab Z, Antal I, Kiss J, Szendroi M, Csoka M, Kovacs G (2011). Good prognosis of localized osteosarcoma in young patients treated with limb-salvage surgery and chemotherapy. Pediatr Blood Cancer.

[15] Pakos EE, Nearchou AD, Grimer RJ, Koumoullis HD, Abudu A, Bramer JA, Jeys LM, Franchi A, Scoccianti G, Campanacci D (2009). Prognostic factors and outcomes for osteosarcoma: an international collaboration. Eur J Cancer.

[16] Nathan SS, Healey JH (2012). Demographic determinants of survival in osteosarcoma. Ann Acad Med Singap.

[17] Janeway KA, Barkauskas DA, Krailo MD, Meyers PA, Schwartz CL, Ebb DH, Seibel NL, Grier HE, Gorlick R, Marina N (2012). Outcome for adolescent and young adult patients with osteosarcoma: a report from the Children’s Oncology Group. Cancer.

[18] Pruksakorn D, Phanphaisarn A, Arpornchayanon O, Uttamo N, Leerapun T, Settakorn J (2015). Survival rate and prognostic factors of conventional osteosarcoma in Northern Thailand: a series from Chiang Mai University Hospital. Cancer Epidemiol.

[19] Hung GY, Yen HJ, Yen CC, Chen WM, Chen PC, Wu HT, Chiou HJ, Chang WH, Hsu HE (2015). Experience of pediatric osteosarcoma of the extremity at a single institution in Taiwan: prognostic factors and impact on survival. Ann Surg Oncol.

[20] Wang B, Tu J, Yin J, Zou C, Wang J, Huang G, Xie X, Shen J (2015). Development and validation of a pretreatment prognostic index to predict death and lung metastases in extremity osteosarcoma. Oncotarget.

[21] Aggerholm-Pedersen N, Maretty-Nielsen K, Keller J, Baerentzen S, Schroder H, Jorgensen PH, Hansen BH, Nielsen OS, Safwat A (2015). The importance of standardized treatment in high-grade osteosarcoma: 30 years of experience from a hospital-based database. Acta Oncol.

[22] Berner K, Hall KS, Monge OR, Weedon-Fekjaer H, Zaikova O, Bruland OS. Prognostic factors and treatment results of high-grade osteosarcoma in norway: a scope beyond the “classical” patient. Sarcoma. 2015;2015:14. Article ID 516843. 10.1155/2015/516843. 10.1155/2015/516843 PMC4346701 25784831

[23] Bielack SS, Kempf-Bielack B, Delling G, Exner GU, Flege S, Helmke K, Kotz R, Salzer-Kuntschik M, Werner M, Winkelmann W (2002). Prognostic factors in high-grade osteosarcoma of the extremities or trunk: an analysis of 1,702 patients treated on neoadjuvant cooperative osteosarcoma study group protocols. J Clin Oncol.

[24] Edge SB, Byrd DR, Compton CC, Fritz AG, Greene FL, Trotti A (2010). AJCC cancer staging manual.

[25] Bacci G, Longhi A, Versari M, Mercuri M, Briccoli A, Picci P (2006). Prognostic factors for osteosarcoma of the extremity treated with neoadjuvant chemotherapy: 15-year experience in 789 patients treated at a single institution. Cancer.

[26] Ferrari S, Bertoni F, Mercuri M, Picci P, Giacomini S, Longhi A, Bacci G (2001). Predictive factors of disease-free survival for non-metastatic osteosarcoma of the extremity: an analysis of 300 patients treated at the Rizzoli Institute. Ann Oncol.

[27] Faisham WI, Mat Saad AZ, Alsaigh LN, Nor Azman MZ, Kamarul Imran M, Biswal BM, Bhavaraju VM, Salzihan MS, Hasnan J, Ezane AM, et al. Prognostic factors and survival rate of osteosarcoma: A single-institution study. Asia Pac J Clin Oncol. 2017;13:e104–10. 10.1111/ajco.12346 25870979

[28] Duchman KR, Gao Y, Miller BJ (2015). Prognostic factors for survival in patients with high-grade osteosarcoma using the Surveillance, Epidemiology, and End Results (SEER) Program database. Cancer Epidemiol.

